# Co-culture of human pancreatic islets with human adipose-derived stromal/stem cells can improve islet quality in vitro

**DOI:** 10.1186/1758-5996-7-S1-A249

**Published:** 2015-11-11

**Authors:** Bianca Marmontel de Souza, Liana Paula Abreu da Silva, Ana Paula Bouças, Jakeline Rheinheimer, Fernanda dos Santos de Oliveira, Ciro Paz Portinho, Bruno Paiva dos Santos, Nance Beyer Nardi, Melissa Camassola, Luis Henrique Canani, Andrea Carla Bauer, Daisy Crispim

**Affiliations:** 1 Universidade Federal do Rio Grande do Sul, Porto Alegre, Brazil

## Background

In patients with unstable type 1 diabetes mellitus (T1D), allogeneic pancreatic islet transplantation is a therapeutic option to restore insulin secretion and improve metabolic control. However, the success of islet transplantation is dependent on the number and quality of isolated islets. It is known that the inflammatory environment related to the donor's brain-death (BD) and the stress induced during islet isolation reduces islet quality. Adipose-derived stromal/stem cells (ASC) are multipotent cells that release several trophic factors with anti-inflammatory and cytoprotective actions. Thus, in vitro co-culture of islets with ASCs may improve islet quality isolated from BD-donors, attenuating inflammation and apoptosis.

## Objectives

To evaluate the effect of co-culture of human pancreatic islets with human ASCs in an indirect contact system has on the improvement of islet quality in vitro.

## Materials and methods

Human islets were isolated according to the method described by Ricordi et al. (1989). ASCs were isolated from lipoaspirates using the protocol established by Zuk et al. (2001). All patients (for adipose tissue samples) and donor's relatives (for pancreas) signed an informed consent form. Islets were cultivated alone or in indirect contact with ASCs using inserts in 6-well plates for 24h, 48h and 72h. Viability was determined by FDA/PI staining and function was evaluated by glucose stimulated-insulin secretion (GSIS). Gene expressions of HIF1α (anti-hypoxia), HMOX1 (cytoprotector) and XIAP (anti-apoptotic) were evaluated by RT-qPCR.

## Results

Islets co-cultured with ASCs demonstrated higher viability and GSIS indexes after 72h than islets cultured alone (viability: 95.2±2.8 vs 89.5±3.6; P=0.046; GSIS: 1.6±0.7 vs 1.0±0.1; P=0.01). Co-cultured islets seem to have increased HIF1α expression as compared to islets alone [6.7±5.9 vs 3.2±0.9 arbitrary units (AU), P=0.058]. Moreover, XIAP expression was increased in islets alone as compared to the 72h co-culture condition (4.3±0.5 vs 3.0±0.5 AU; P=0.031). HMOX1 expression was similar between groups.

## Conclusion

These preliminary results indicate that co-culture of pancreatic islets with ASCs promotes an improvement on islet quality. Thus, co-culture prior to clinical transplantation may be a viable option for improving islet quality and, consequently, the success of islet transplantation.
